# Supplementary material for: Assessment of Narrow-Band Imaging Algorithm for Video Capsule Endoscopy Based on Decorrelated Color Space for Esophageal Cancer: Part II, Detection and Classification of Esophageal Cancer
Source: Cancers (Basel). 2024 Jan 29;16(3):572. doi: 10.3390/cancers16030572 (PMC10854620; doi:10.3390/cancers16030572)
Supplement: Supplementary file 1 [file cancers-16-00572-s001.zip › cancers-2837801-supplementary-done.pdf]

cancers

MDPI

Article

Assessment of Narrow Band Imaging Algorithm for Video Capsule Endoscopy Based on Decorrelated Color Space for Esophageal Cancer: Part II, Detection and Classification of Esophageal Cancer: Supplementary Material

Yu-Jen Fang [1,2,†], Chien-Wei Huang [3,4,†], Riya Karmakar [5], Arvind Mukundan [5], Yu-Ming Tsao [5], Kai-Yao Yang [3,*] and Hsiang-Chen Wang [5,6,7,*]

1 Department of Internal Medicine, National Taiwan University Hospital, Yun-Lin Branch, No. 579, Sec. 2, Yunlin Rd., Dou-Liu 64041, Taiwan
2 Department of Internal Medicine, National Taiwan University College, Department of Internal Medicine, National Taiwan University College of Medicine, No. 1, Jen Ai Rd., Sec. 1, Taipei 10051, Taiwan
3 Department of Gastroenterology, Kaohsiung Armed Forces General Hospital, 2, Zhongzheng 1st.Rd., Lingya District, Kaohsiung 80284, Taiwan
4 Department of Nursing, Tajen University, 20, Weixin Rd., Yanpu Township, Pingtung County 90741, Taiwan
5 Department of Mechanical Engineering, National Chung Cheng University, 168, University Rd., Min Hsiung, Chia Yi 62102, Taiwan
6 Department of Medical Research, Dalin Tzu Chi Hospital, Buddhist Tzu Chi Medical Foundation, No. 2, Minsheng Road, Dalin, Chia Yi 62247, Taiwan
7 Director of Technology Development, Hitspectra Intelligent Technology Co., Ltd., 4F, No. 2, Fuxing 4th Rd., Qianzhen District, Kaohsiung 80661, Taiwan
* Correspondence: yangkaiyao@gmail.com (K.-Y.Y.); hcwang@ccu.edu.tw (H.-C.W.)
† These authors contributed equally to this work.

Citation: Fang, Y.-J.; Huang, C.-W.; Karmakar, R.; Mukundan, A.; Tsao, Y.-M.; Yang, K.-Y.; Wang, H.-C. Assessment of Narrow-Band Imaging Algorithm for Video Capsule Endoscopy Based on Decorrelated Color Space for Esophageal Cancer: Part II, Detection and Classification of Esophageal Cancer. *Cancers* **2024**, *16*, 572. https://doi.org/10.3390/cancers16030572

Academic Editors: Michaela Cellina and Filippo Pesapane

Received: 7 January 2024
Revised: 26 January 2024
Accepted: 26 January 2024
Published: 29 January 2024

Copyright: © 2024 by the authors. Licensee MDPI, Basel, Switzerland. This article is an open access article distributed under the terms and conditions of the Creative Commons Attribution (CC BY) license (https://creativecommons.org/licenses/by/4.0/).

Abstract: This article provides supplementary materials for the article “Detection and Classification of Esophageal Cancer using Narrow Band Imaging based on Decorrelated Color Space combined with YOLOv5”. Section 1 gives an overview of the Yolo architecture and the loss functions. Section 2 briefly describes on the equations that was used for calculating the sensitivity, specificity, F1-score, accuracy and precision of the Yolo model. Section 3 shows the equations that were used for NBI and WLI Image Comparisons. Section 4 provides the detailed values of the results of image Comparison in terms of SSIM, entropy and PSNR and the section 5 illustrates the simulated NBI images with the corresponding WLI images.

Keywords: Narrow Band Imaging, Hyperspectral Imaging, Decorrelated Color Space, Peak Signal to Noise Ration, Structural Similarity Index Metric, Entropy, YOLO

S1. YOLO Architecture and Loss Functions

The network is mainly composed of backbone, neck, and head. Backbone is a convolutional neural network layer, neck is a feature extraction layer, and Head uses the GIOU loss function.

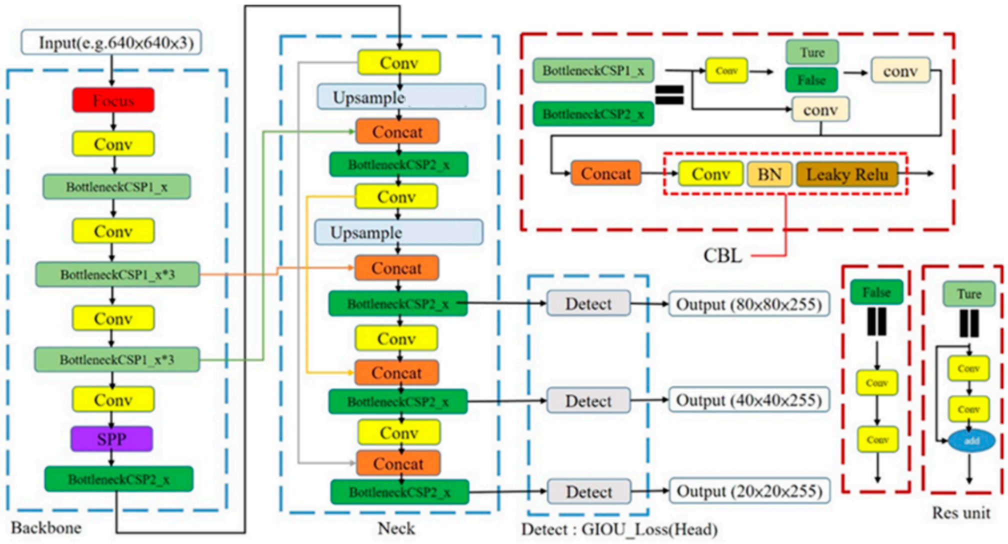

Figure S1. YOLOv5 network architecture

The batch size was set to 16 and the epoch was set to 800 while the model was set to stop training when there is not much improvement in the loss, precision and recall. However, the best model was produced when the epoch was 486. The precision confidence curve shows the tradeoff between precision for different threshold. A high area under the curve represents both high recall and high precision, where high precision relates to a low false positive rate, and high recall relates to a low false negative rate.

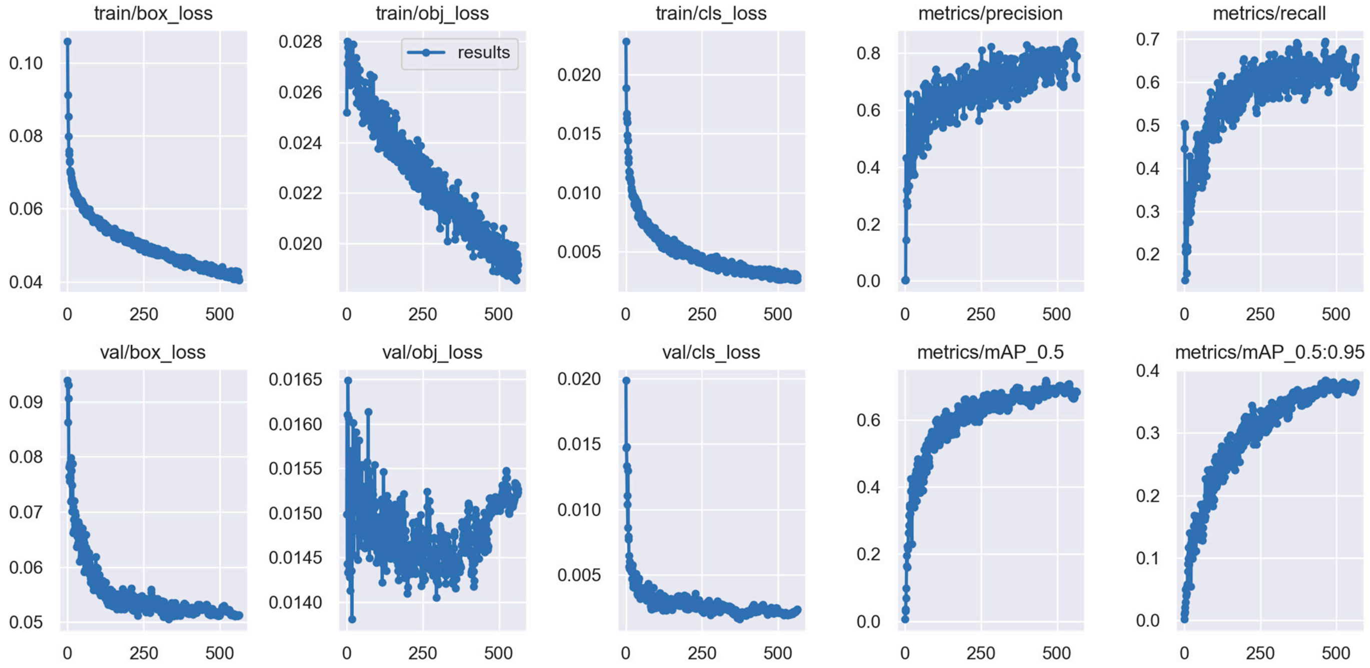

Figure S2. Convergence of loss functions for training set of WLI images and precision, recall, and average precision.

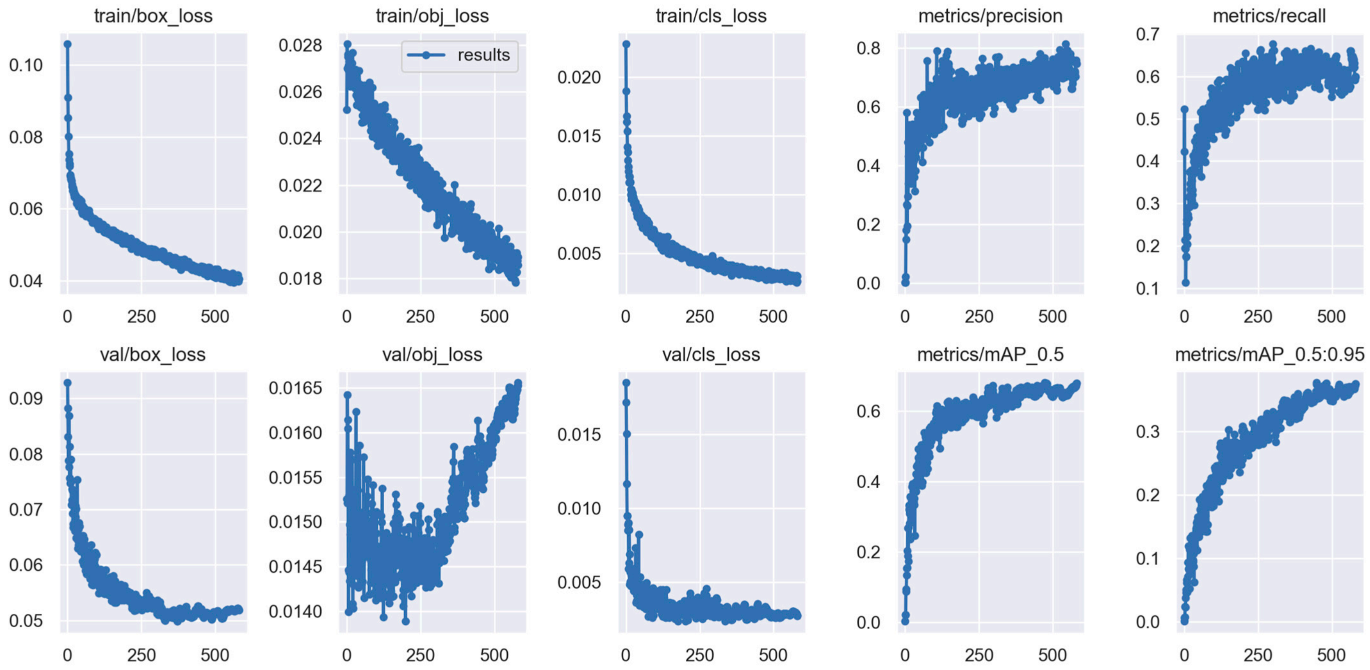

Figure S3. NBI image training set loss functions and convergence of precision, recall, and mean precision

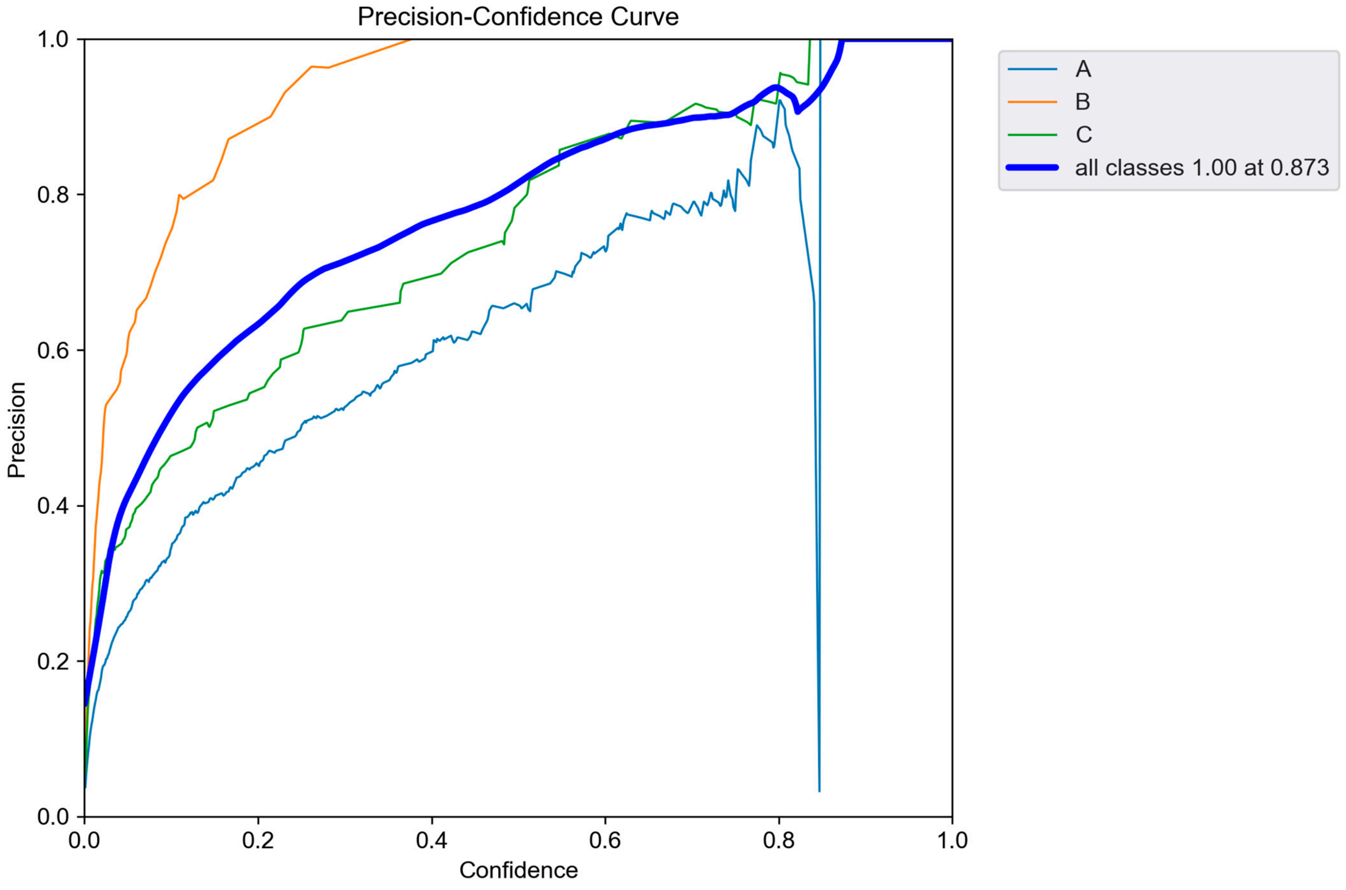

Figure S4. Precision-Confidence curve for the WLI image dataset

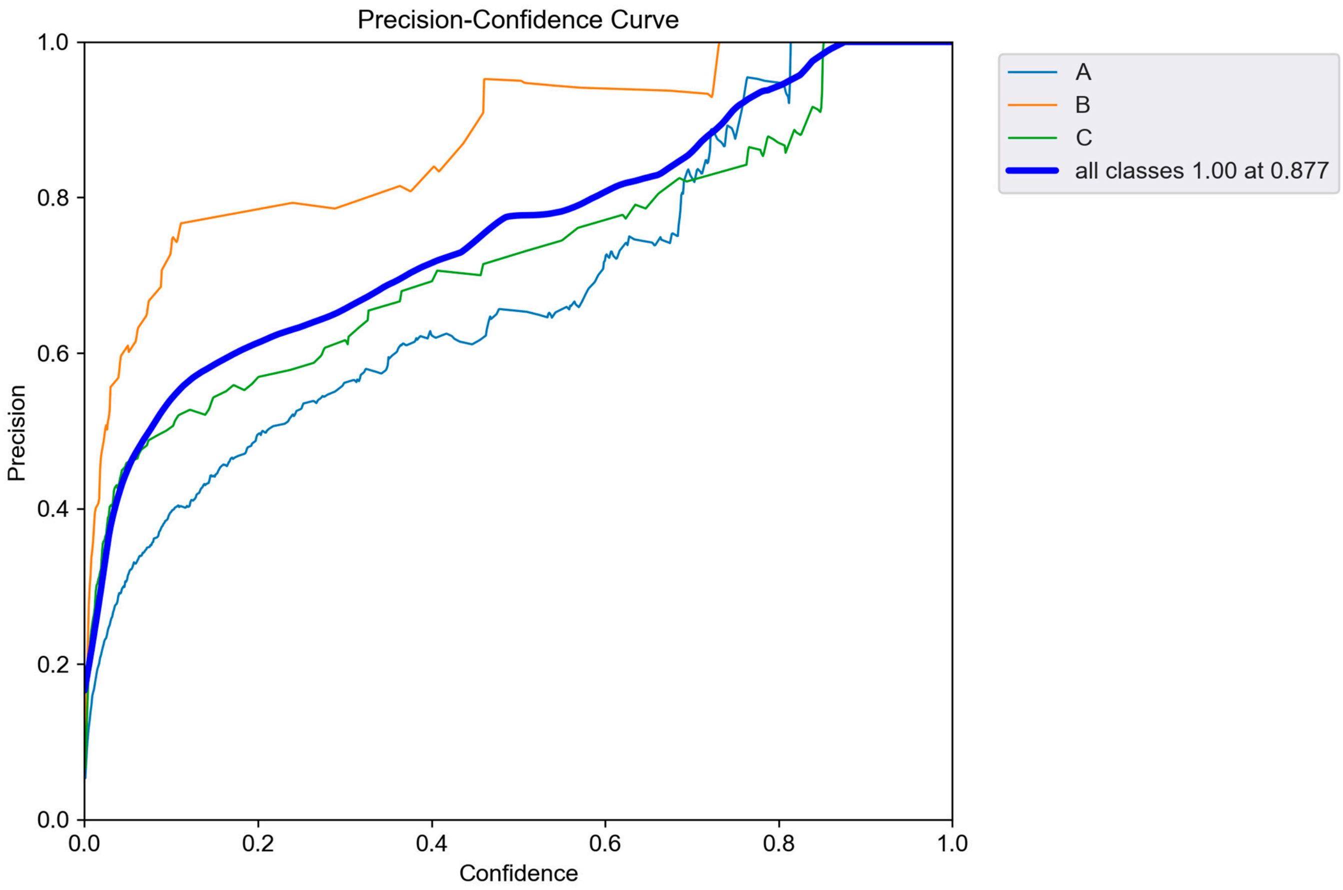

Figure S5. Precision-Confidence curve for the NBI image dataset

S2. Equations for Classification Indicators

The sensitivity indicates how well the model can detect symptoms of esophageal cancer. The accuracy value indicates the proportion of esophageal cancer and actual cancer symptoms in the model's diagnosis. F1-score is a harmonic mean, and it can be used as a rough indicator of the model performance. Kappa value can be used to evaluate the consistency between prediction and pathological analysis results to evaluate the feasibility of prediction tools. Its value is between −1 and 1 and often has a threshold of 0.6. AP is a commonly used evaluation index for object detection, and the overall sensitivity and accuracy are used to quantify the overall performance of a prediction model.

$$\text{Precision} = \frac{\text{TP}}{\text{TP+FP}} \quad \text{(S1)}$$

$$\text{Sensitivity} = \frac{\text{TP}}{\text{TP+FN}} \quad \text{(S2)}$$

$$\text{Specificity} = \frac{\text{TN}}{\text{TN+FP}} \quad \text{(S3)}$$

$$\text{F1} = \frac{2}{\frac{1}{\text{Precision}}+\frac{1}{\text{Recall}}} \quad \text{(S4)}$$

$$\text{Accuracy} = \frac{\text{TP+TN}}{\text{TP+TN+FP+FN}} \quad \text{(S5)}$$

S3. Equations for Image Comparisons

The entropy of an image is defined as follows:

$$-\sum_{i=0}^{n-1} p_i \log_b p_i$$

where *n* is the number of gray levels (256 for 8-bit images), *pi* is the probability of a pixel having gray level *i*, and *b* is the base of the logarithm function. Notice that the entropy of an image is rather different from the entropy feature extracted from the GLCM (Gray-Level Co-occurrence Matrix) of an image.

The structural similarity index measure (SSIM) is a method for predicting the perceived quality of digital television and cinematic pictures, as well as other kinds of digital images and videos. SSIM is used for measuring the similarity between two images. It can be represented by the following equation:

$$\mathrm{SSIM}(x,y) = \frac{(2\mu_x\mu_y + c_1)(2\sigma_{xy} + c_2)}{(\mu_x^2 + \mu_y^2 + c_1)(\sigma_x^2 + \sigma_y^2 + c_2)}$$

μ_x = the pixel sample mean of x;

μ_y = the pixel sample mean of y;

C_1 and C_2 = variables to stabilize the division with weak denominator

K_1 = 0.01 and K_2 = 0.03

σ_{xy} = covariance of x and y

L = dynamic range of pixel values

σ_x^2 = covariance of x

σ_y^2 = covariance of y

PSNR is most easily defined via the mean squared error (*MSE*). Given a noise-free *m*×*n* monochrome image *I* and its noisy approximation *K*, *MSE* is defined as:

$$MSE = \frac{1}{m\,n}\sum_{i=0}^{m-1}\sum_{j=0}^{n-1}[I(i,j) - K(i,j)]^2.$$

The PSNR (in dB) is defined as:

$$\begin{aligned} PSNR &= 10 \cdot \log_{10}\left(\frac{MAX_I^2}{MSE}\right) \\ &= 20 \cdot \log_{10}\left(\frac{MAX_I}{\sqrt{MSE}}\right) \\ &= 20 \cdot \log_{10}(MAX_I) - 10 \cdot \log_{10}(MSE). \end{aligned}$$

Here, MAX_I is the maximum possible pixel value of the image. When the pixels are represented using 8 bits per sample, this is 255. More generally, when samples are represented using linear PCM with *B* bits per sample, MAX_I is $2^B - 1$.

S4. Results of Image Comparison

Table S1. Results of PSNR comparison of each image in Olympus and VCE

PSNR Comparision		
# image	Olympus	VCE
1	28.3033	28.4234
2	27.5851	27.4912
3	28.6736	28.012
4	27.656	29.008
5	27.6027	29.0184
6	27.782	28.8618
7	28.4473	28.0175
8	27.8109	28.5319
9	27.5827	28.3681
10	27.5073	27.9671
11	27.6241	27.8602
12	27.646	27.8653
13	29.6058	27.7043
14	28.028	27.9716
15	29.7888	27.6491
16	29.2247	27.6757
17	27.7772	27.5595
18	28.4386	27.7358
19	27.7769	27.8191
20	28.1393	27.7566
Average	28.15003	28.06484

Table S2. Results of Entropy comparison of each image in Olympus and VCE

Entropy Comparision				
# Images	Olympus		Vce	
	WLI	NBI	WLI	NBI
1	7.4692	7.17155	6.43501	6.950931
2	7.696849	7.55283	6.55779	6.95735
3	7.64243	7.37639	6.43802	6.88498
4	7.58609	7.34965	6.47801	6.93397
5	7.46084	7.08171	6.52072	7.00907
6	7.51781	7.33392	6.66476	7.01961
7	7.34168	6.84817	6.49488	7.1053
8	7.41681	7.10808	6.5661	7.19722
9	7.46113	7.24866	6.53207	7.17257
10	7.50348	7.24452	6.86103	7.19379
11	7.41662	7.18721	6.99237	7.26331
12	7.42663	7.16701	7.06938	7.27016
13	7.52686	7.42154	7.10936	7.27413
14	7.74991	7.62265	7.10993	7.25245
15	7.63398	7.35181	7.03728	7.18075
16	7.48843	7.31261	7.04451	7.1778
17	7.53016	7.21741	7.07349	7.19443
18	7.44276	7.20028	7.10469	7.16334
19	7.52783	7.19222	7.13437	7.19183
20	7.46343	7.29484	7.14958	7.19768
Average	7.515147	7.264154	6.818668	7.129534
Difference	3.455%		4.559%	

Table S3. Results of SSIM comparison of each image in Olympus and VCE

SSIM Comparision		
# Number	Olympus	VCE
1	98.37	92.65
2	96.91	92.86
3	98.6	92.49
4	99.7	92.23
5	99.56	91.74
6	99	92.15
7	99.64	91.17
8	99.55	92.12
9	99.66	92.1
10	99.35	93.42
11	96.11	94.09
12	99.3	94.2
13	97.12	94.24
14	99.45	94.21
15	98.3	94.12
16	99.32	94.07
17	97.42	94.07
18	96.11	94.1
19	96.72	94.16
20	99	94.2
Average	98.4595	93.2195

S5. Simulated NBI Images

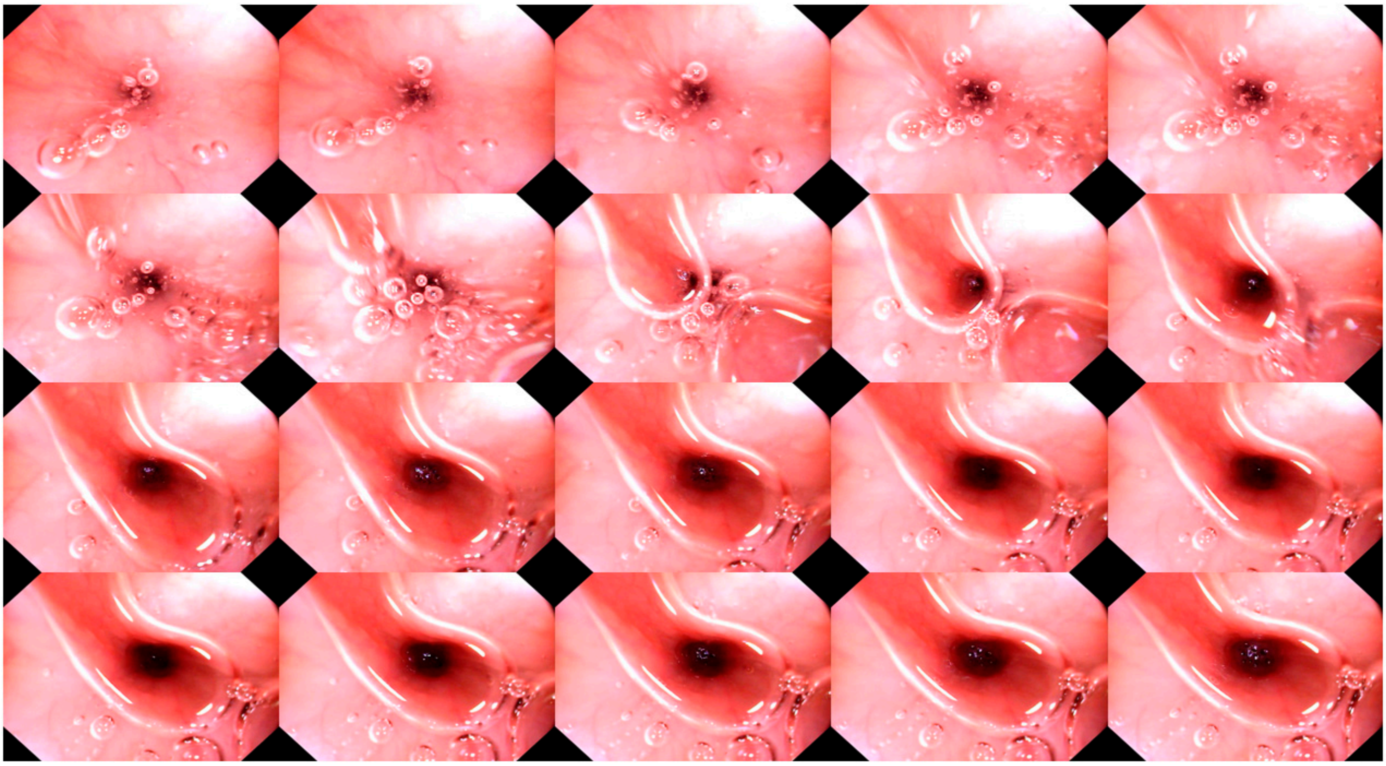

Figure S6. 20 Randomly chosen images of WLI in VCE

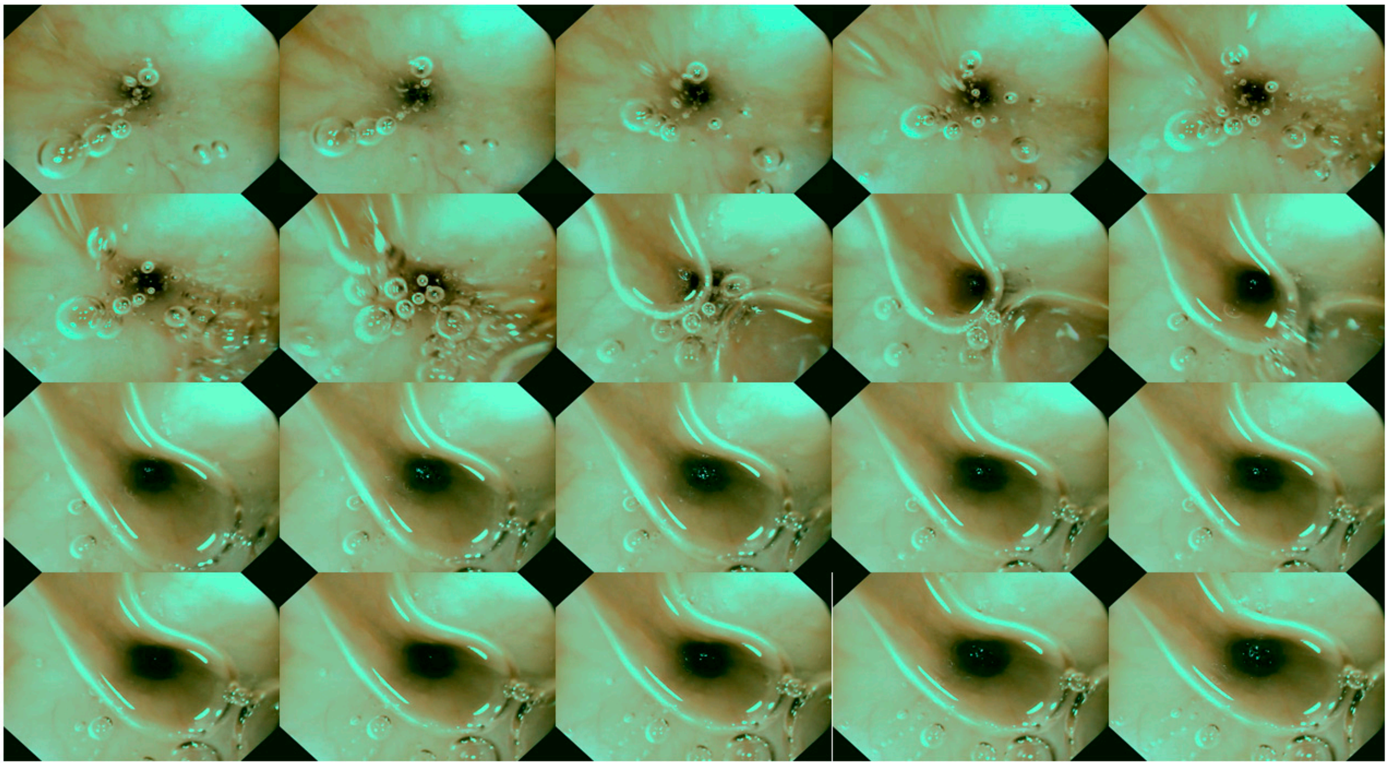

Figure S7. 20 Randomly chosen images of NBI in VCE

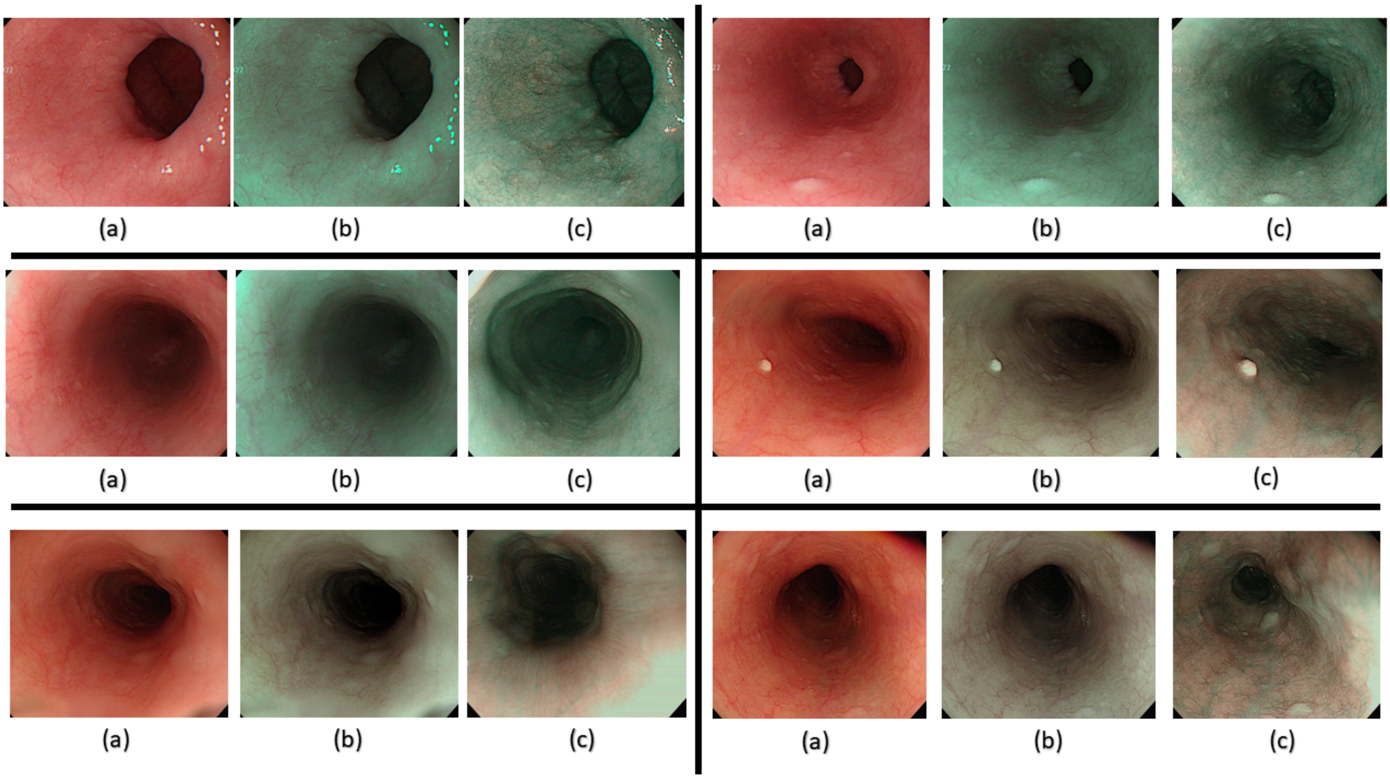

Figure S8. 6 Randomly choses WLI images, simulated NBI images and a similar original NBI in Olympus endoscope. (a) Olympus WLI images. (b) simulated NBI image and (c) a similar NBI image from Olympus

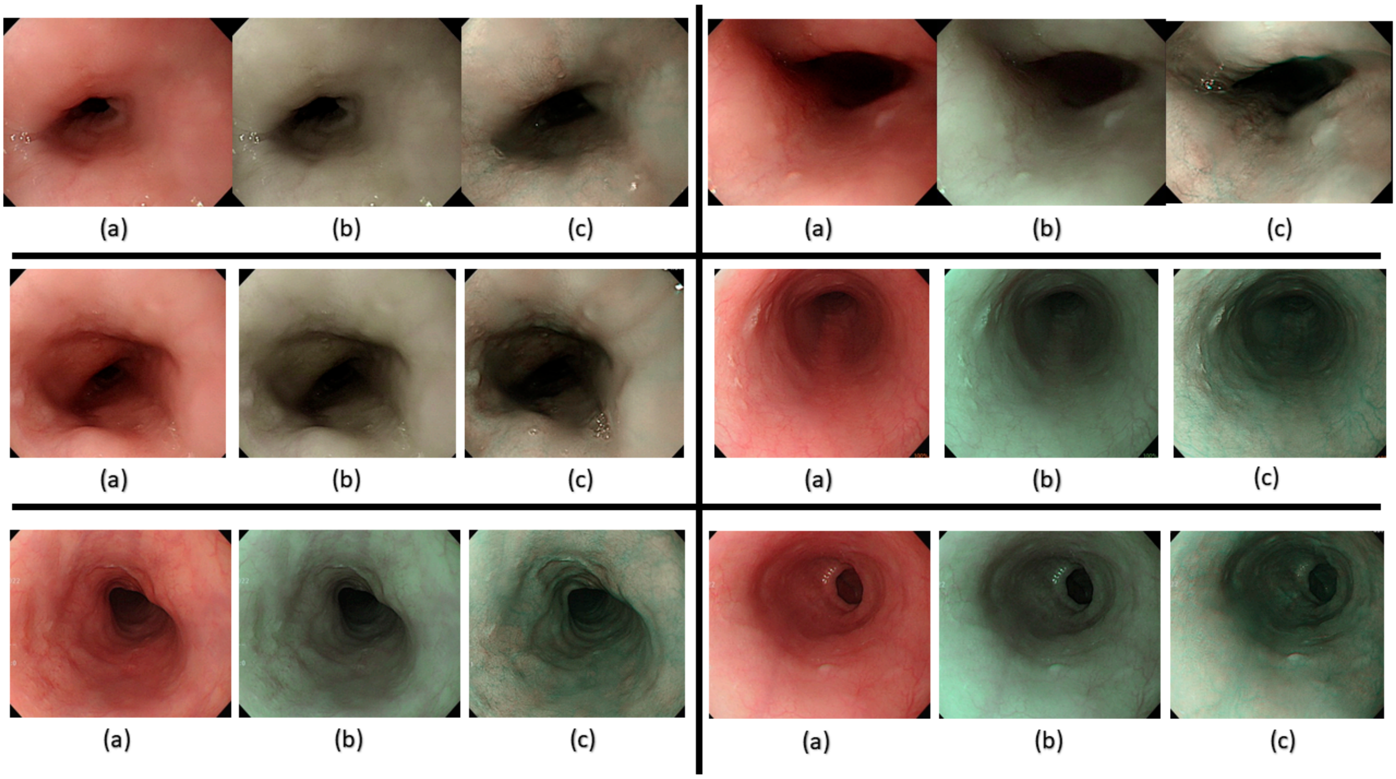

Figure S9. 6 Randomly choses WLI images, simulated NBI images and a similar original NBI in Olympus endoscope. (a) Olympus WLI images. (b) simulated NBI image and (c) a similar NBI image from Olympus

S6. Hyperparameters in Yolo

Learning rate: The learning rate is a hyperparameter that governs the magnitude of parameter updates in a neural network during training. The selection of a learning rate is crucial in defining the speed at which the network reaches the optimal answer and its ability to generalize to unfamiliar material. A learning rate of 0.01 has been maintained.

Weight decay: It is a regularization approach employed in deep learning to mitigate overfitting by including a penalty term into the loss function. The penalty term is directly proportional to the square of the size of the weights in the network. The weight decay hyperparameter governs the intensity of the penalty term and dictates the extent to which the weights are contracted towards zero. The weight decay for this investigation was maintained at a value of 5x10-4.

Intersection over Union (IoU): It is a criterion used to distinguish between a genuine positive and a false positive for a predicted bounding box of an item. The IoU threshold represents the least level of overlap that must exist between the predicted bounding box and the ground truth bounding box for the prediction to be classified as a true positive. Thus, in this analysis, a specific range of 0.30 is maintained as the optimal value.

Focal Loss: It is is a modified version of the cross-entropy loss function that specifically targets the issue of class imbalance in object detection tasks. In the typical cross-entropy loss, all classes are assigned identical weights, which may result in suboptimal performance when there is an abundance of easily classifiable samples and just a small number of challenging ones. The focal loss function provides a reduced weight to simple examples and an increased weight to challenging ones, enabling the model to prioritize its attention on the more difficult instances. Consequently, the decision has been made to retain 0 as the focus loss.

Augmentation: The augmentation techniques, such as image translation, image rotation, and image scale, have been assigned a value of 0.2, while all other augmentation techniques relating to color and shear-flip have been assigned a value of 0 for this study.

Author Contributions: Conceptualization, H.-C.W and A.M.; data curation, Y.-M.T and A.M.; formal analysis, A.M.; funding acquisition, H.-C.W.; investigation, Y.-M.T and A.M.; methodology, H.-C.W and A.M.; project administration, Y.-M.T, A.M. and H.-C.W.; resources, H.-C.W and A.M.; software, Y.-M.T and A.M.; supervision, Y.-M.T and H.-C.W.; validation, Y.-M.T and A.M.; writing—original draft, A.M.; writing—review and editing, A.M. and H.-C.W. All authors have read and agreed to the published version of the manuscript.

Funding: This research was supported by the National Science and Technology Council, The Republic of China, under the grants NSTC 111-2221-E-194-007. This work was financially/partially supported by the Advanced Institute of Manufacturing with High-tech Innovations (AIM-HI) and the Center for Innovative Research on Aging Society (CIRAS) from The Featured Areas Research Center Program within the framework of the Higher Education Sprout Project by the Ministry of Education (MOE) in Taiwan.

Institutional Review Board Statement: The study was conducted in accordance with the Declaration of Helsinki and approved by the Institutional Review Board.

Informed Consent Statement: Not applicable.

Data Availability Statement: Not Applicable.

Conflicts of Interest: The authors declare no conflict of interest.

Disclaimer/Publisher's Note: The statements, opinions and data contained in all publications are solely those of the individual author(s) and contributor(s) and not of MDPI and/or the editor(s). MDPI and/or the editor(s) disclaim responsibility for any injury to people or property resulting from any ideas, methods, instructions or products referred to in the content.
